# Practice Patterns Vary Among Orthopedic, Plastic, and General Surgeons Resecting Soft Tissue Tumors of the Extremities and Pelvis

**DOI:** 10.1002/jso.70147

**Published:** 2025-11-30

**Authors:** Jon‐Luc Poirier, Spencer M. Richardson, Adam M. Knox, L. Daniel Wurtz, Christopher D. Collier

**Affiliations:** ^1^ Department of Orthopedic Surgery Indiana University Health, Indiana University School of Medicine Indianapolis IN USA

## Abstract

**Background:**

Resection of extremity soft tissue tumors is common and frequently performed by orthopedic, plastic, and general surgeons. It is unknown if tumor location, Preoperative workup, and Postoperative care varies by specialty, which is the aim of this study.

**Methods:**

A retrospective review was performed of 4,223 soft tissue tumors resected from the extremities and pelvis within a large single‐state health system between 2009 and 2019. A more detailed cross‐sectional review was performed on 450 tumors resected in 2016. Demographic and tumor characteristics, surgeon specialty (orthopedic, plastic, general), Preoperative workup (imaging, biopsy), and Postoperative management were collected and analyzed.

**Results:**

General surgeons were more likely to resect tumors superficial to fascia (82.1%), compared to plastic and orthopedic surgeons (53.7% and 27.9%). Orthopedic surgeons were more likely to resect malignant tumors (28.2%) than plastic and general surgeons (14.0% and 4.5%). 16.3% of tumors resected by general surgeons had either Preoperative MRI or tissue diagnosis, compared to 42.6% for plastic surgeons and 90.5% for orthopedic surgeons (*p* < 0.001). Of the tumors resected by general surgeons without Preoperative MRI or tissue diagnosis, 2.6% were malignant. Finally, Postoperative documentation of neurovascular status, range of motion, and referral to physical therapy were more likely performed by orthopedic surgeons (all *p* < 0.001).

**Conclusion:**

Practice patterns vary significantly among orthopedic, plastic, and general surgeons treating soft tissue tumors of the extremities and pelvis. These findings highlight the need for multidisciplinary engagement and standardization of treatment algorithms and training practices across the various surgical specialties.

## 1 Introduction

Soft tissue tumors are common, most often benign, and frequently referred to orthopedic, plastic, or general surgeons for resection [1, 2]. Little is known about the practice patterns across these surgical specialties in their management of soft tissue tumors. Prior comparisons have assessed large national databases to identify differences in perioperative outcomes and case volumes between surgical specialties treating soft tissue sarcoma [3] or soft tissue tumors [4]. Shannon et al [3] observed no difference in perioperative morbidity and mortality between general and orthopedic surgeons over a period from 2008 to 2017. General surgeons performed the majority of sarcoma resections during this time period (69.4%), however, their specialty‐specific sarcoma resection declined over 35% during this time period, potentially signaling a shift in practice patterns. Moreover, their methodology did not allow for more detailed examination of pre‐ or Postoperative management. For example, these studies [3, 4] did not assess differences in Preoperative imaging, which may be critical in reducing unplanned resection of malignant histologies, as a lack of Preoperative imaging has been identified in the majority of unplanned soft tissue sarcoma resections which may ultimately lead to increased number of surgical interventions for the patient [5, 6]. Additionally, the influence of training on Postoperative physical examination and referral to physical therapy has not been explored, which could impact long‐term range of motion and musculoskeletal function.

Considering the potential contribution of surgeon specialty and associated training to practice patterns in the management of soft tissue tumors, we asked: (1) Does the anatomic tumor location differ by resecting surgeon specialty? (2) How does the Preoperative management of patients differ by surgeon specialty? (3) Are there specialty‐specific differences in Postoperative management of soft tissue tumor patients?

## 2 Patients and Methods

### 2.1 Study Design and Setting

This is a retrospective study of clinical data derived from a large single‐state healthcare system consisting of 15 hospitals including academic, community, and rural sites. All soft tissue tumors resected between 2009 and 2019 of the upper extremity, lower extremity, and pelvis were identified using CPT code (23071, 23073, 23075, 23076, 23077, 23078, 24071, 24073, 24075, 24076, 24077, 24079, 25071, 25073, 25075, 25076, 25077, 25078, 26111, 26113, 26115, 26116, 26117, 26118, 27043, 27045, 27047, 27048, 27049, 27059, 27327, 27328, 27329, 27337, 27339, 27364, 27615, 27616,27618, 27619, 27632, 27634, 28039, 28047, 28041, 28043, 28045, 28046). These years were selected to maximize the availability of electronic medical record information and minimize the influence of any aberrant trends during the COVID‐19 pandemic in 2020.

### 2.2 Data Source

The data is abstracted by a single reviewer from a single institution's electronic medical record (EMR) using CPT code identification.

### 2.3 Inclusion and Exclusion Criteria

**Figure 1:**
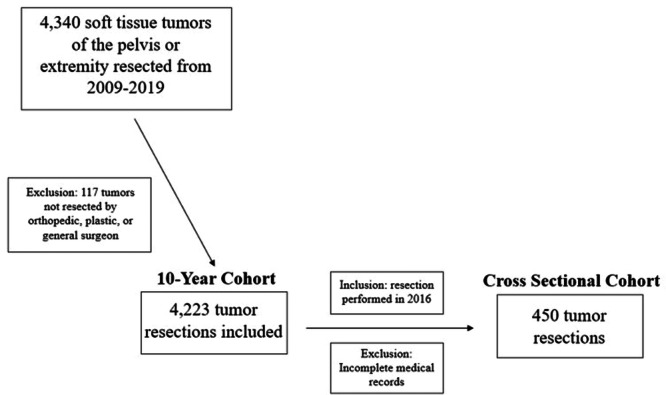
Work flow for cross‐sectional population identification.

We identified 4,341 soft tissue tumors of the extremity or pelvis resected between 2009 and 2019. Resections with incomplete clinical data or performed by surgeons who completed residencies outside of orthopedic, plastic, and general surgery (117 patients) were excluded leaving 4,223 soft tissue tumor resections for inclusion. A cohort of 450 tumors resected in 2016 with complete medical records was identified for a more detailed cross‐sectional analysis of practice patterns by surgeon specialty (Figure 1). 2016 was chosen for the cross‐sectional arm of this study to maximize EMR data available and to allow for follow‐up preceding the COVID‐19 pandemic.

### 2.4 Extracted Data and Outcomes

For the entire study population, tumor data identified by CPT code included tumor size, depth, histological behavior (inferred from excision *vs.* radical excision codes), and anatomical location. Surgeon specialty was identified by self‐designation upon review of surgeon biography. For the 2016 cohort, data collection also included whether Preoperative imaging was obtained, type of Preoperative imaging, and whether a Preoperative tissue biopsy was performed. Postoperative variables collected included referral to physical therapy and whether a neurovascular exam and/or range of motion (ROM) assessment of the adjacent joint was performed and documented within 3 months of operative intervention. Final follow‐up was determined based off the final documentation by the operative provider or supporting advanced practice provider.

### 2.5 Ethical Approval

This study was exempted after review by the Institutional Review Board (IRB #14464), given the absence of direct patient contact and minimal risk of patient harm. All data was anonymized following abstraction at the earliest possible juncture.

### 2.6 Statistical Analysis, Study Size

Descriptive statistics were performed to assess tumor characteristics and case volumes by surgical specialty. Chi‐squared analysis was used to determine the difference in pre‐ and post‐ operative practice patterns by surgeon specialty. A two‐tailed p value of less than 0.05 was considered statistically significant. Statistical analysis was performed using SPSS Statistics 28 (IBM, Armonk, NY).

## 3 Results

**Figure 2:**
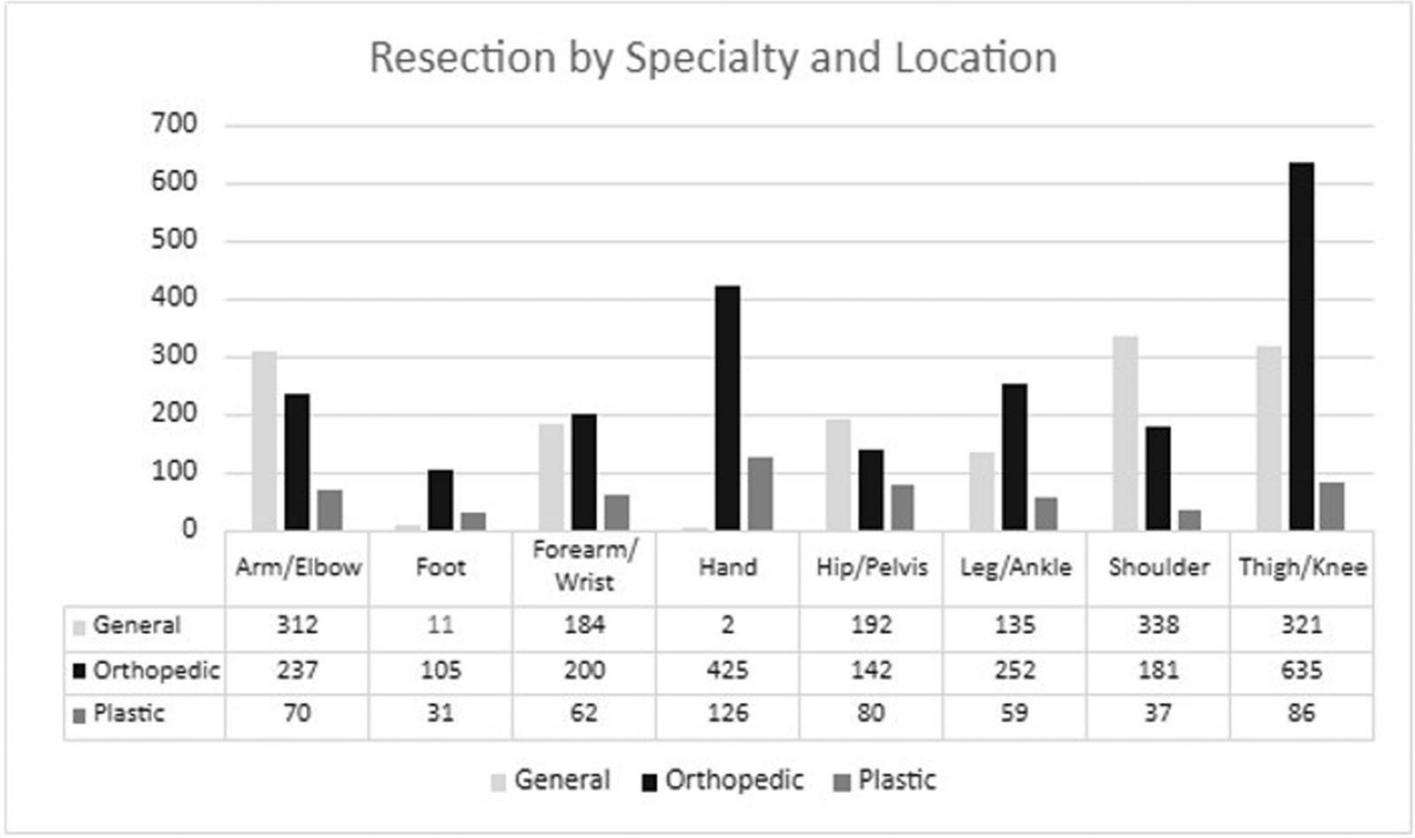
Tumor resections by location and surgical specialty.

4,223 soft tissue tumors of the extremity or pelvis met the inclusion criteria for the comparative study of practice patterns between the years 2009‐2019 (Figure 1). Of these, 2,177 (51.6%) resections were performed by orthopedic surgeons, 1495 (35.4%) by general surgeons, and 551 (13.0%) by plastic surgeons (Table 1). General surgeons most often resected tumors superficial to the fascial plane (82.1%) compared to plastic and orthopedic surgeons (53.7% and 27.9%, respectively). Orthopedic surgeons proportionally performed the most resections of thigh/knee tumors (29.2%, 635/2177), leg/ankle tumors (11.6%, 252/2177), and hand tumors (19.5%, 425/2177), (Figure 2). General surgeons performed more tumor resections in the upper and lower extremity limb girdles, shoulder tumors (22.6%, 338/1495) and hip/pelvis (12.4% 192/1495). Orthopedic surgeons proportionately resected more malignant tumors (28.2%) than plastic and general surgeons (14.0% and 4.5%, respectively).

**Table 1:** Case numbers by surgeon type and tumor depth and behavior.

Variable	Orthopedic	Plastic	General
**Tumor Depth**			
Superficial	608 (27.9%)	296 (53.7%)	1227 (82.1%)
Deep	955 (43.9%)	178 (32.3%)	201 (13.4%)
Malignant	614 (28.2%)	77 (14%)	67 (4%)
**Tumor Behavior**			
Benign	1563 (71.8%)	474 (86%)	1428 (85.5%)
Malignant	614 (28.2%)	77 (14%)	67 (4.5%)
**Total Tumors by Specialty**	2177	551	1495

Within the 2016 cross‐sectional cohort, 450 patients met inclusion criteria (Figure 1). Orthopedic surgeons resected the majority of tumors (231, 51.3%) versus plastic (47, 10.4%) and general surgeons (172, 38.3%) (Table 2). Orthopedic surgeons performed more Preoperative imaging (90.5% of patients) than did plastic or general surgeons (27.7% vs. 22.7% respectively, *p* < 0.001) (Table 3). Preoperative magnetic resonance imaging (MRI) was obtained in 71.0% of the resections performed by orthopedic surgeons, as compared to 12.8% of plastic surgery resections and 9.9% of general surgery resections (*p* < 0.001). Resections performed by plastic surgeons had the highest rate of Preoperative tissue biopsy (34.0%) compared to orthopedic and general surgeons (29.4% vs. 3.5% respectively, *p* < 0.001). 90.5% of tumor resections performed by orthopedic surgeons were done following either tissue diagnosis or MRI imaging, as compared to plastic surgery (42.6%) and general surgery (16.3%) (*p* < 0.001). No orthopedic or plastic surgeons resected a malignant tumor without preoperative imaging or tissue diagnosis. Within the general surgery group, 4 (2.3%) tumors of malignant histology were resected without pre‐operative tissue diagnosis or MRI imaging. Within 3 months of operative intervention, 56.3% of patients underwent a documented neurovascular assessment by their treating orthopedic surgeon, as compared to 10.6% of plastic surgery patients, and 3.5% of general surgery patients (*p* < 0.001). Within the 3‐month post‐operative period, adjacent joint range of motion assessment was performed and documented in 63.2% of orthopedic patients, 10.6% of plastic surgery patients, and 6.4% of general surgery patients (*p* < 0.001). Documented physical therapy referral rates were low overall, at 24.2% of orthopedic patients, 10.6% of plastic surgery patients, and 0.6% of general surgery patients (*p* < 0.001).

**Table 2:** Descriptive characteristics of study population. Surgical specialty, tumor behavior and depth, and care location are reported.

Patient Demographic Table	
**Age in years, median (IQR)**	50.2 (40.0‐61.3)
**Specialty**	
Orthopedic	231 (51.3%)
Plastic	47 (10.4%)
General	172 (38.3%)
**Pathology**	
Benign	375 (83.3%)
Malignant	75 (16.7%)
**Greatest Diameter in cm, median (IQR)**	4 (2‐7)
**Depth**	
Superficial	253 (56.2%)
Deep	144 (32.0%)
N/a	53 (11.8%)
**Treating Facility**	
Urban/Academic	232 (51.5%)
Community	134 (29.8%)
Rural	84 (18.7%)
**Reresection**	26 (5.7%)
**Follow Up: median, [IQR]**	
Orthopedic	20, [13–42]
Plastic	11, [0, 63]
General	12, [12–28]

**Table 3:** Results from chi‐squared analysis of pre‐ and Postoperative practice patterns. Proportions are listed in parentheses, with significance at *p* < 0.001 for all variables.

Variable	Orthopedic	Plastic	General	*p* value
**Preoperative Imaging**	211 (90.5%)	13 (27.7%)	39 (22.7%)	< 0.001
**Preoperative MRI**	164 (71.0%)	6 (12.8%)	17 (9.9%)	
**Preoperative Tissue Biopsy**	68 (29.4%)	16 (34.0%)	6 (3.5%)	< 0.001
**Tumors Resected with either MRI or Tissue Diagnosis**	209 (90.5%)	20 (42.6%)	28 (16.3%)	< 0.001
**Tumors Resected Without Preoperative MRI or Tissue Diagnosis**				
Benign	22 (100.0%)	27 (100%)	140 (97.2%)	
Malignant	0 (0.0%)	0 (0.0%)	4 (2.8%)	
**Postoperative Neurovascular Assessment**	130 (56.3%)	5 (10.6%)	6 (3.5%)	< 0.001
**Postoperative ROM Assessment**	146 (63.2%)	5 (10.6%)	11 (6.4%)	< 0.001
**PT Referral**	56 (24.2%)	5 (10.6%)	1 (0.6%)	< 0.001
**Total Patient**	231	47	172	

## 4 Discussion

### 4.1 Multiple Surgical Specialties Resect Soft Tissue Tumors of the Extremities

Canter et al. have reported on a wide range of surgical specialists treating soft tissue tumors of the extremities and found that while general surgeons accounted for 47% of the surgeons included, they only performed 9.4% of the deep or malignant tumor resections [4]. Additionally, there were similar rates of subcutaneous (superficial) resections seen among all specialties. In our study, orthopedic surgeons resected more malignant soft tissue tumors (*n* = 614) than did plastic (*n* = 77) or general (*n* = 67) surgeons. Our data also suggests that the majority of resections performed by general surgeons were superficial, 82.1%. Conversely, superficial resections comprised only 27.9% of orthopedic resections.

This trend may suggest that orthopedic surgeons more commonly operate deep to the fascia in the extremities as compared to general surgeons. In a 2008‐2017 analysis, the National Surgical Quality Improvement Program (NSQIP) of extremity soft tissue sarcoma reported a higher proportion of resections performed by general surgery during their study period; however, the proportion of resections performed by general surgery decreased by 35.2% during the study period, and resections by orthopedic surgeons increased [7]. An anatomic feature that may also partially skew resection frequencies reported is tumors of the hand. Our data suggest that orthopedic and plastic surgeons proportionately operate more in the distal upper extremity, accounting for 19.5% and 22.5% of their total tumor volume, respectively. This is contrasted with hand resections accounting for only 0.1% of all general surgery resections. While potentially influenced by surgeon preferences, this may also suggest that referral patterns may influence the frequency of tumor resection in a given anatomic area. It stands to reason that tumors more peripherally located (hand, foot, forearm/wrist, leg/ankle) in the extremity may be more likely to be referred to an orthopedic or plastic surgery provider as compared to a general surgeon, who is potentially less commonly associated by referring providers with operating on these anatomic areas. In our study, a higher proportion of tumor resections for orthopedic surgeons (45.1%) and or plastic surgeons (50.5%) were peripherally located (hand, foot, forearm/wrist, leg/ankle), as compared to general surgeons (22.1%).

### 4.2 Orthopedic Surgeons Perform More Preoperative Workup of Soft Tissue Tumors Than Plastic or General Surgeons

We found that orthopedic surgeons perform more Preoperative workup of soft tissue tumors than do plastic or general surgeons. Preoperative MRI can reliably differentiate between benign and potentially malignant lesions as well as inform the surgeon on the relation of nearby anatomic structures to the tumor [5, 8, 9, 10]. In our study, orthopedic surgeons obtained a preoperative MRI on 71.0% of their cases, as compared to 12.8% of plastic surgeons and 9.9% of general surgeons. Plastic and orthopedic surgeons also resected a higher proportion of biopsy‐confirmed tumors (34.0% and 29.4%, respectively) versus general surgery (3.5%). Together, orthopedic surgeons resected tumors after more extensive preoperative characterization (MRI and/or tissue diagnosis) than did plastic or general surgeons on average (90.5% vs. 42.6% and 16.3% respectively). This trend is primarily driven by higher utilization of MRI, which may reflect a difference in surgeon training. Moreover, orthopedic and plastic surgeons more often utilize MRI in the treatment of non‐neoplastic entities such as nervous, ligamentous, or bony injuries that general surgeons may not routinely encounter.

While Canter et al. [4] found no difference in morbidity and mortality between surgeon specialties in the resection of all soft tissue tumor behaviors, Mesko et al. [5] described potential risk factors for adverse outcomes in the resection of malignant histology. In their study of soft tissue sarcomas, Mesko et al. found a significant difference in MRI usage among patients who underwent a negative margin resection versus those who underwent an incomplete resection (91.0% vs. 42.0%, *p* < 0.05). Lack of preoperative biopsy was also associated with lower rates of complete resection (16.0%) versus those with a preoperative biopsy (85.0%, *p* < 0.05). While existing literature suggests there is no difference in adverse outcomes among different surgical specialties in the resection of all soft tissue tumors (benign and malignant), differences in preoperative work‐up may affect outcomes in the malignant tumor population.

The Accreditation Council for Graduate Medical Education (ACGME) stipulates a minimum of 10 orthopedic oncology cases during orthopedic residency [11], and most orthopedic surgeon residencies include a dedicated orthopedic oncology rotation. Plastic surgery is required to resect 8 neoplasms of the upper extremity, whereas general surgery residents are not mandated a minimum number of musculoskeletal tumor cases [12, 13]. 159/231 (68.8%) of orthopedic tumor resections were performed by musculoskeletal oncologists, contrasted with 9/172 (5.2%) general surgery tumors being resected by oncology‐trained surgeons. Thus, the training and experiences gained from mandated musculoskeletal oncology cases during residency and fellowship may, in part, drive more extensive tumor workups. Moreover, many patients referred to orthopedic oncologists are referred out of concern for malignancy. Therefore, a potential for bias to suspect malignancy may exist in the musculoskeletal oncologist's approach to tumor characterization and preoperative planning, thereby leading to increased rates of MRI and tissue biopsy.

Siegel et. al identified resection deep to fascia, no preoperative imaging, and MRI reports that do not include sarcoma within their differentials as risk factors for unplanned sarcoma resection [14]. Although MRI has been identified as an important diagnostic tool [15], this latter finding highlights the importance of clinical experience and context abstracted from surgical training, supplementing diagnostic workup and the interpretation of imaging studies. A retrospective review published found a higher rate of local tumor recurrence in patients who underwent unplanned resection followed by secondary resection as compared to those who underwent planned resection (21.4% vs. 13.7%) [7]. They also found 48/51(94.1%) required wide reexcision, and 3/51 (5.9%) patients required limb amputation. Local recurrence has been associated with metastatic disease and reduced overall survival [16]. These studies emphasize the long‐term sequelae and potential harms of unplanned resection. Although a relatively small proportion of general surgery resections in our study were unplanned malignancies, they represent a potentially avoidable occurrence with a more extensive work‐up.

### 4.3 The Postoperative Management of Soft Tissue Tumor Resections Differs by Specialty

Postoperative trends by surgeon specialty differed, most notably in the documented physical exam. Orthopedic surgeons were most likely to assess postoperative neurovascular status and adjacent joint ROM. This may in part be due to their ACGME case requirements, with at least 1000 required surgeries performed in the upper, lower extremity, and pelvis. Thus, musculoskeletal examination may be more ingrained as part of the postoperative patient management, much in the same way an abdominal exam may be second nature to a general surgeon. Of note, the ACGME requires that plastic surgery residents perform 122 surgical cases related to the musculoskeletal system in the upper extremity. The influence of neurovascular exam on postoperative outcomes is, however, unclear, thus limiting our ability to abstract clinically meaningful inferences from this data. Referral to physical therapy following soft tissue tumor resection is less common, with orthopedic surgeons referring the highest proportion of patients. This may be driven by the high incidence of physical therapy referrals seen in common non‐oncologic orthopedic surgeries such as arthroplasty and fracture care. However, there is discordance even among orthopedists and physical therapists over the need for a formal physical therapy referral, with many surgeons finding home exercise adequate [17]. Given this and the paucity of data characterizing patient functionality following soft tissue tumor resection, interpretation and abstraction of our data remains limited. However, the lowest rate of therapy referral, observed in the general surgery cohort, may also be due in part to the lack of ACGME‐required musculoskeletal case during general surgery residency.

### 4.4 Limitations

Limitations of our study include the retrospective nature and data collection from a single statewide health care system. As our study was able to look at soft tissue mass resections on a provider‐specific level, a prospective study involving all surgeons in our statewide system would be logistically challenging and could induce bias, such as the Hawthorne Effect (i.e., observer effect). Surgeons may alter their workup and treatment of a soft tissue mass if known to be participating in a study. We believe the distribution of our statewide healthcare system in rural, urban, and metropolitan areas and a 10‐year study period allows generalization of our comparative study findings to other institutions, though we certainly recognize that practice and referral patterns may be institution‐specific. Another limitation of the 10‐year comparative cohort of our study is the limited clinical information, as variables were collected based on CPT procedure coding alone. Third, the pre‐ and post‐operative management of the entire 2009‐2019 cohort was not further characterized due to the demands of accessing 4223 medical records of varying time frames. We therefore selected the 2016 cohort for the cross‐sectional analysis of surgeon practice to ensure comprehensive EMR documentation and to avoid the impact of the COVID‐19 pandemic during follow‐up, though restricting this analysis could have led to some bias. Fourth, tumor size and location in relation to fascia were not controlled for when evaluating surgeon's preoperative workup and post‐operative exam, follow‐up, and physical therapy referral. It can be argued that smaller and superficial tumors do not require biopsy, preoperative imaging, or postoperative physical therapy, which could bias our results. However, it is worth noting that preoperative imaging was still obtained in 90.5% of masses resected by orthopedic surgeons. Finally, we did not include outcome measures such as reoperation, postoperative complications, patient satisfaction, or measures of postoperative function. Based on the results of this study, we think these would be interesting areas of future research.

## 5 Conclusion

We present a large retrospective review of surgical trends and practice patterns in the resection of soft tissue tumors of the extremities and pelvis. The increased case volumes seen in the extremities, deep to the fascial plane, and of malignant histological behavior by orthopedic surgeons may lead to increased clinical familiarity with treatment of malignant tumors. Orthopedic surgeons more commonly obtain preoperative MRI and/or tissue diagnosis than plastic and general surgeons which may lead to more extensively characterized tumors before resection but may also lead to increased healthcare costs and unnecessary imaging. Likely reflective of their focused musculoskeletal training, orthopedic surgeons performed the highest rates of musculoskeletal specific postoperative examination and referral to therapy, although the impact of this on surgical outcome is unclear. In totality, these observations may be due in part to the emphasis placed by the ACGME on musculoskeletal and oncologic requirements during orthopedic and plastic residency and fellowship. Thus, surgical experiences and training garnered during residency may dictate surgeon practice in the resection of soft tissue tumors of the extremity and pelvis. Given our findings, future investigation characterizing the influence of surgeon training and practice patterns on tumor patient outcomes may be warranted.

## Ethics Statement

Ethical approval for this study was waived by Indiana University Institutional Review Board, Indianapolis, IN, USA.

## Conflicts of Interest

Each author certifies that there are no funding or commercial associations (consultancies, stock ownership, equity interest, patent/licensing arrangements, etc.) that might pose a conflict of interest in connection with the submitted article related to the author or any immediate family members.

## Synopsis

This retrospective clinical review of 4,223 soft tissue tumor resections in the extremities assesses trends in practice stratified by surgeon specialty and training. We aim to highlight trends in the surgical management of these tumors so as to work further towards standardizing care for these patients.

## Data Availability

The data that support the findings of this study are available on request from the corresponding author. The data are not publicly available due to privacy or ethical restrictions.
